# Inter-residue through-space scalar ^19^F–^19^F couplings between CH_2_F groups in a protein

**DOI:** 10.5194/mr-6-131-2025

**Published:** 2025-07-14

**Authors:** Yi Jiun Tan, Elwy H. Abdelkader, Iresha D. Herath, Ansis Maleckis, Gottfried Otting

**Affiliations:** 1 ARC Centre of Excellence for Innovations in Peptide and Protein Science, Research School of Chemistry, Australian National University, Canberra, ACT 2601, Australia; 2 Latvian Institute of Organic Synthesis, Aizkraukles 21, 1006 Riga, Latvia

## Abstract

Using cell-free protein synthesis, the protein G B1 domain (GB1) was prepared with uniform high-level substitution of leucine by (2 S ,4 S )-5-fluoroleucine (FLeu1), (2 S ,4 R )-5-fluoroleucine (FLeu2), or 5,5^′^-difluoroleucine (diFLeu). ${}^{19}F$ nuclear magnetic resonance (NMR) spectra showed chemical shift ranges spanning more than 9 ppm . Through-space scalar ${}^{19}F{\text{–}}^{19}F$ couplings between ${\mathrm{CH}}_{2}F$ groups arising from transient fluorine–fluorine contacts are readily manifested in [ ${}^{19}F$ , ${}^{19}F$ ]-TOCSY spectra. The ${}^{19}F$ chemical shifts correlate with the three-bond ${}^{1}H$ – ${}^{19}F$ couplings ( ${}^{3}J_{\mathrm{HF}}$ ), confirming the γ -gauche effect as the predominant determinant of the ${}^{19}F$ chemical shifts of the ${\mathrm{CH}}_{2}F$ groups. Different ${}^{3}J_{\mathrm{HF}}$ couplings of different ${\mathrm{CH}}_{2}F$ groups indicate that the rotation of the ${\mathrm{CH}}_{2}F$ groups can be sufficiently restricted in different protein environments to result in the preferential population of a single rotamer. The ${}^{3}J_{\mathrm{HF}}$ couplings also show that ${\mathrm{CH}}_{2}F$ groups populate the different rotameric states differently in the 5,5^′^-difluoroleucine residues than in the monofluoroleucine analogues, showing that two ${\mathrm{CH}}_{2}F$ groups in close proximity influence each other's conformation. Nonetheless, the ${}^{19}F$ resonances of the $C^{\delta 1}H_{2}F$ and $C^{\delta 2}H_{2}F$ groups of difluoroleucine residues can be assigned stereospecifically with good confidence by comparison with the ${}^{19}F$ chemical shifts of the enantiomerically pure fluoroleucines. ${}^{1}H{\text{–}}^{19}F$ nuclear Overhauser effects (NOEs) observed with water indicate hydration with sub-nanosecond residence times.

## 1 Introduction

Proteins made with global substitution of a single amino acid type by a selectively fluorinated analogue greatly facilitate their analysis by ${}^{19}F$ NMR spectroscopy (Sharaf and Gronenborn, 2015). Structural perturbations caused by the fluorine substitutions can be kept to a minimum if a single fluorine atom is installed in a methyl group as the resulting ${\mathrm{CH}}_{2}F$ group has the freedom to respond to the increased spatial requirement of the C–F moiety by preferential population of those rotamers that are most readily accommodated by the chemical environment. Recently, we showed that the *Escherichia coli* peptidyl–prolyl isomerase B (PpiB), which contains five leucine residues, can be produced with high-level uniform substitution of leucine for (2 S ,4 S )-5-fluoroleucine (FLeu1), (2 S ,4 R )-5-fluoroleucine (FLeu2), or 5,5^′^-difluoroleucine (diFLeu; Fig. [Fig F1]) using cell-free protein synthesis (Tan et al., 2024). As demonstrated by X-ray crystal structures, the structural perturbations caused by these amino acid substitutions were minimal (Frkic et al., 2024a). Furthermore, the ${}^{3}J_{\mathrm{HF}}$ coupling constants were inversely correlated with the ${}^{19}F$ chemical shifts in a first experimental confirmation of the γ -gauche effect predicted by Oldfield and co-workers based on quantum calculations (Feeney et al., 1996). In the structure of PpiB, the leucine residues are isolated from each other. In contrast, the three leucine residues of GB1 are arranged such that methyl groups of neighbouring leucine residues can make van der Waals contacts (Fig. [Fig F2]). This situation may produce through-space scalar $J_{\mathrm{FF}}$ ( ${}^{\mathrm{TS}}J_{\mathrm{FF}}$ ) couplings. Scalar couplings through non-bonded interactions are common for atoms containing free electron pairs (Hierso, 2014), and a ${}^{\mathrm{TS}}J_{\mathrm{FF}}$ coupling of 21 Hz has been reported between two fluorotryptophan residues in a protein (Kimber et al., 1978).

**Figure 1 F1:**
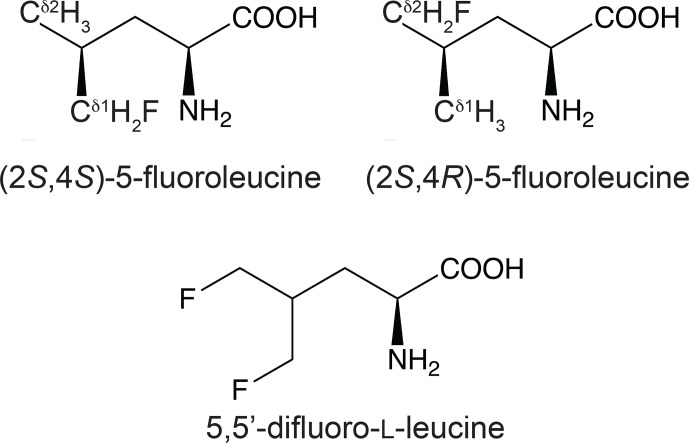
Chemical structures of the fluorinated leucine analogues used in the present work. (2 S ,4 S )-5-fluoroleucine (where fluorine is on the $\delta_{1}$ carbon), (2 S ,4 R )-5-fluoroleucine (where fluorine is on the $\delta_{2}$ carbon), and 5,5^′^-difluoro-L-leucine are referred to in the following as FLeu1, FLeu2, and diFLeu, respectively.

### 1.1 Experimental procedures

### 1.2 Fluorinated leucine analogues

Initially, the fluorinated leucine analogues FLeu1 and FLeu2 (Fig. [Fig F1]) were synthesised following published protocols (Moody et al., 1994; August et al., 1996; Charrier et al., 2004). diFLeu with and without ${}^{2}H$ substitutions was synthesised as described (Maleckis et al., 2022). Subsequently, FLeu1, FLeu2, and diFLeu were obtained as HCl salts from Enamine (Ukraine).

### 1.3 Expression vectors

Expression vectors were based on pETMCSI (Neylon et al., 2000) and constructed with a C-terminal ${\mathrm{His}}_{6}$ tag following a TEV cleavage site. The amino terminus was preceded by the 5^′^-nucleotide sequence of the T7 gene 10 to ensure high expression yields, which added the hexapeptide MASMTG. The full nucleotide and amino acid sequences are shown in Table S1 in the Supplement.

### 1.4 Protein expression

All protein samples were expressed by continuous exchange cell-free protein synthesis (CFPS) following an established protocol (Apponyi et al., 2008; Ozawa et al., 2012). The gene of the GB1 construct was PCR-amplified with eight-nucleotide overhangs to generate circularised DNA suitable for use in CFPS (Wu et al., 2007). Leucine was omitted when preparing the acid-soluble amino acid mixture. The fluoroleucine of interest was added from an aqueous stock solution to the outer buffer at a final concentration of 4 mM . The pH of the outer buffer was adjusted to 7.5. The CFPS reaction was conducted at 30 °C for 16 h using 1 mL inner reaction mixture of S30 cell extract made from the *E. coli* BL21 strain and 10 mL outer buffer.

### 1.5 Protein purification

Proteins were purified using a 1 mL Ni–NTA gravity column (GE Healthcare, USA) equilibrated with buffer A (50 mM Tris– HCl , pH 7.5, 300 mM NaCl ) using buffer B (same as buffer A but with 10 mM imidazole) for washing and buffer C (same as buffer A but with 300 mM imidazole) for elution. The purified proteins were dialysed overnight against a storage buffer (50 mM HEPES, pH 7.5, 100 mM NaCl ) and concentrated using an Amicon centrifugal ultrafiltration tube with a molecular weight cut-off of 3 kDa .

### 1.6 Protein mass spectrometry

Intact protein mass analysis was performed using an Orbitrap Elite Hybrid Ion Trap–Orbitrap mass spectrometer equipped with an UltiMate 3000 UHPLC (Thermo Scientific, USA). The protein samples were injected via an Agilent ZORBAX SB-C3 Rapid Resolution HT Threaded Column using a 5 %–80 % gradient of acetonitrile with 0.1 % formic acid. The data were collected in positive ion mode. The protein masses were obtained by deconvolution using the Xtract function in the Qual Browser software tool of the program Xcalibur 3.0.63 (Thermo Fisher Scientific, USA).

### 1.7 Protein NMR conditions

All ${}^{19}F$ NMR spectra were measured at 25 °C on a 400 MHz Bruker Avance NMR spectrometer equipped with a SmartProbe, allowing ${}^{19}F$ detection with ${}^{1}H$ decoupling. The protein solutions were in 90 % $H_{2}O$ / 10 % $D_{2}O$ with 20 mM MES buffer, pH 6.5, and 100 mM NaCl or with 50 mM HEPES buffer, pH 7.5, and 100 mM NaCl . All spectra reported of GB1 made with diFLeu were recorded in MES buffer. All spectra reported of GB1 made with FLeu2 were recorded in HEPES buffer. All spectra reported of GB1 made with FLeu1 were recorded in HEPES buffer unless indicated otherwise. 0.1 mM trifluoroacetate (TFA) was added as an internal reference and calibrated to - 75.25 ppm .

### 1.8 Results

### 1.9 Protein yields and purity

Up to 2.7 mg of protein was obtained from 1 mL inner reaction mixture of the CFPS setup (Table S2 in the Supplement). The amino acid sequence of native GB1 contains three leucine residues, and the additional TEV cleavage site present in our constructs adds a fourth leucine residue. Intact protein mass spectrometry indicated that the predominant species contained fluorinated leucine analogues at all four sites. The species containing three or two fluorinated leucine analogues were also detected, but their intensity indicated that the chance of canonical leucine at any of the four sites was below 10 % (Fig. S1 in the Supplement). Mass spectra of GB1 produced with diFLeu in the presence of some canonical leucine delivered the natural protein as the main species followed by protein containing single leucine-for-diFLeu substitutions, illustrating the strong preference of the *E. coli* leucyl-tRNA synthetase for L-leucine over diFLeu (Fig. S2 in the Supplement). Complete exclusion of L-leucine from the CFPS reaction could not be achieved due to amino acid impurities in the S30 extract.

### 1.10 Protein stability

Thermal denaturation measured by circular dichroism at 216 nm showed that the melting temperatures the GB1 samples made with fluorinated leucine analogues ranged between about 66 and 72 °C , i.e. 9–15° lower than for the wild-type protein (Fig. S3 in the Supplement), indicating that the presence of ${\mathrm{CH}}_{2}F$ groups decreases the stability of the protein.

### 1.11 1D ${}^{19}F$ NMR spectra

Figure [Fig F3] shows the 1D ${}^{19}F$ NMR spectra of the GB1 variants produced with diFLeu (GB1-d), FLeu1 (GB1-1), or FLeu2 (GB1-2). In addition, Fig. [Fig F3]b shows the spectrum of GB1 produced with diFLeu in the presence of canonical L-leucine (GB1-dd). The 1D NMR spectra resolve the signals of all fluorine atoms.

The ${}^{19}F$ chemical shifts were insensitive to the buffer and pH but very sensitive to the immediate chemical environment. A striking illustration are the very different chemical shifts observed in GB1-d when the sample was prepared with the addition of L-leucine to produce samples predominantly containing single diFLeu residues (GB1-dd; Fig. [Fig F3]a and b). Comparison of the high-field and low-field ends of the spectra of GB1-dd and GB1-d shows that minor peaks observed for the GB1-d sample correspond to main peaks observed with GB1-dd and vice versa. The minor peaks in Fig. [Fig F3]a can thus be attributed to a small amount of canonical leucine in the protein preparation. Conversely, the minor peaks in the spectrum of GB1-dd appear to correspond to peaks of the fully fluorinated GB1-d sample, although the only minor species present in significant amounts contains no more than two diFLeu residues. This indicates that the presence of a second diFLeu residue is sensed only if it is in the immediate neighbourhood. Position 7 features two neighbouring leucine sites (Fig. [Fig F2]), yet the $C^{\delta 1}H_{2}F$ group seems to sense predominantly a single neighbour, while the chemical shift of the $C^{\delta 2}H_{2}F$ group is less well conserved between the major species in GB1-dd and the minor species in GB1-d (Fig. [Fig F3]a and b).

**Figure 2 F2:**
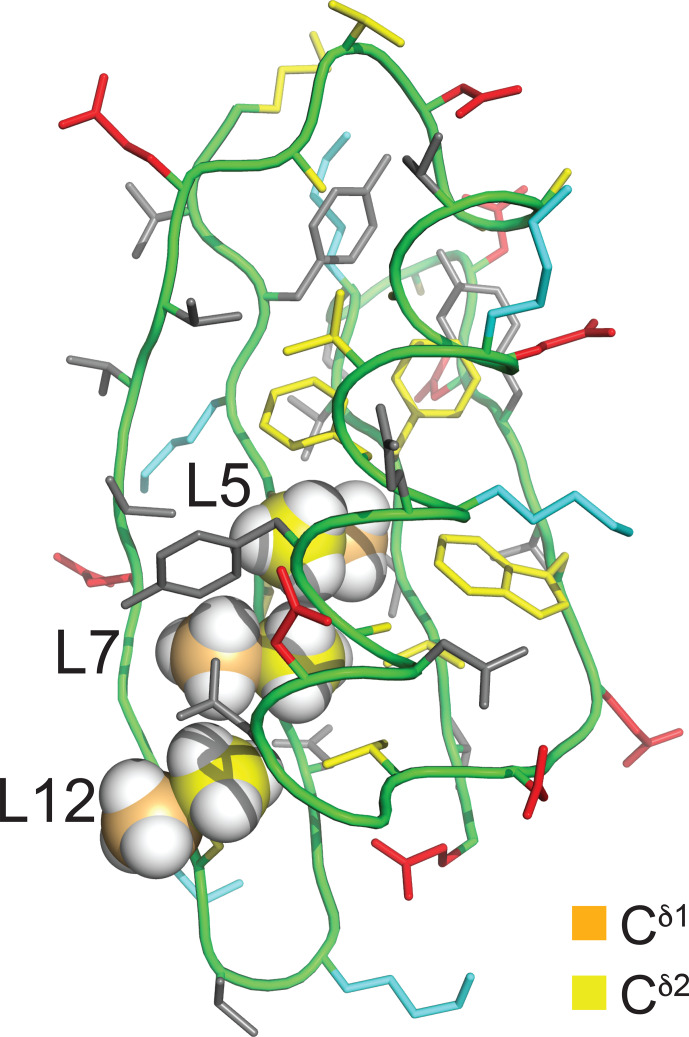
Solution structure of GB1 (PDB ID 3GB1; Kuszewski et al., 1999). The methyl groups of Leu5, Leu7, and Leu12 are shown in a space-filling representation with the $\delta_{1}$ - and $\delta_{2}$ -carbon atoms in orange and yellow, respectively. The side chain of Leu5 is inaccessible to the solvent, whereas the $C^{\delta 1}H_{3}$ groups of Leu7 and Leu12 are partially and highly accessible, respectively. The colour code of the other amino acids is red for negatively charged, blue for positively charged, grey for hydrophilic, and yellow for hydrophobic amino acids.

**Figure 3 F3:**
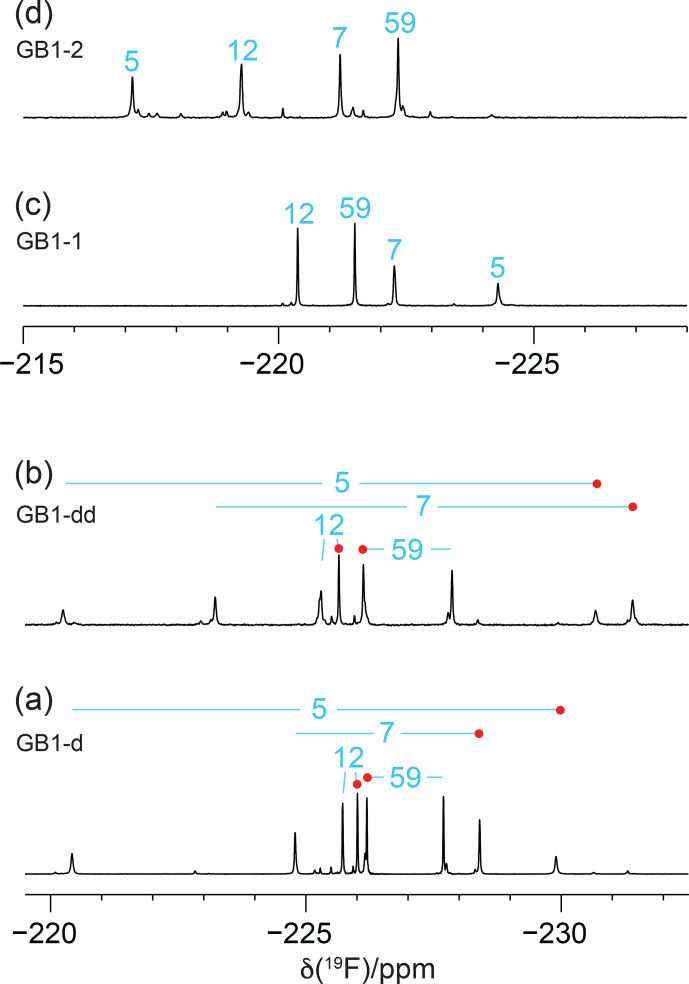
1D ${}^{19}F$ NMR spectra of GB1 made with fluorinated leucine analogues using FLeu1 to produce GB1-1, FLeu2, for GB1-2 and diFLeu for GB1-d and GB1-dd. All spectra were recorded with ${}^{1}H$ decoupling during acquisition using a 0.5 s recovery delay between scans. The resonance assignments are indicated by the sequence numbers of the four leucine sites. **(a)** GB1-d prepared with 4 mM diFLeu. Spectrum recorded of a 2 mM protein solution in 20 mM MES buffer, pH 6.5. Red dots mark the resonances assigned to $C^{\delta 1}H_{2}F$ groups. **(b)** GB1-dd prepared with a mixture of 0.5 mM leucine and 4 mM diFLeu. Spectrum recorded of a 4 mM protein solution in 20 mM MES buffer, pH 6.5. Stereospecific assignments are indicated as in **(a)**. **(c)** Spectrum of a 2 mM solution of GB1-1 in 50 mM HEPES, pH 7.5. **(d)** Spectrum of a 2.2 mM solution of GB1-2 in 50 mM HEPES, pH 7.5.

In the case of the GB1-2 sample, minor peaks arose because the FLeu2 amino acid synthesised in house contained about 10 % FLeu1 as an impurity.

In the case of residue 59, which is in the flexible TEV protease recognition site of the C-terminal peptide segment of the protein construct, the chemical shifts are hardly impacted by the rest of the protein, as indicated by their conservation between the spectra of Fig. [Fig F3]a and b. For the diFLeu residue in position 59 (Fig. [Fig F3]a and b), we base the stereospecific assignment on the ${}^{19}F$ chemical shifts observed for this position in GB1-1 and GB1-2 (Fig. [Fig F3]c and d). The stereospecific assignments of the other diFLeu residues were determined by 2D NMR experiments described below. They attributed the high-field signals of residues 5, 7, and 12 to the $C^{\delta 1}H_{2}F$ groups. Notably, the respective signals in GB1-1 are also at higher field than the corresponding signals in GB1-2 (Fig. [Fig F3]c and d).

The $T_{1}$ relaxation times were of the order of 0.3 s , and the full line widths at half height ranged between 7 and 18 Hz . $R_{1\rho }{(}^{19}F)$ relaxation rates of GB1-d indicate that no ${}^{19}F$ signal possesses an intrinsic line width much greater than 12 Hz (Table S3 in the Supplement). For a sample produced with deuterated diFLeu, where all five protons of the isopropyl group are replaced by deuterium (Maleckis et al., 2022), the $R_{1\rho }{(}^{19}F)$ measurements indicated a maximal intrinsic line width of 7 Hz , suggesting that dipolar relaxation by the nearest protons contributes significantly to the ${}^{19}F$ relaxation. The broadest lines were observed for residue 5, the side chain of which is deeply buried in the core of the protein (Fig. [Fig F2]), hence expected to feature the least flexibility and the fastest transverse relaxation rates. Residue 7 is the next-most-buried residue, while the side chain of residue 12 is more highly accessible to the solvent (particularly the $C^{\delta 1}H_{2}F$ group; see Fig. [Fig F2]), and residue 59 is completely solvent-exposed.

Recording the ${}^{19}F$ NMR spectra without decoupling of the ${}^{1}H$ spins revealed broad multiplets with overlap between some of the resonances (Fig. [Fig F4]). The multiplet of each ${\mathrm{CH}}_{2}F$ group is composed of a triplet of doublets due to two-bond couplings, ${}^{2}J_{\mathrm{HF}}$ , within each ${\mathrm{CH}}_{2}F$ group (47 Hz ) and the ${}^{3}J_{\mathrm{HF}}$ coupling with the methine proton of the isopropyl group. ${}^{3}J_{\mathrm{HF}}$ couplings obey a Karplus relationship (Williamson et al., 1968; Gopinathan and Narasimhan, 1971). If the ${}^{3}J_{\mathrm{HF}}$ coupling is small, the envelope of the multiplet appears like a triplet, but ${}^{3}J_{\mathrm{HF}}$ can also be as large as 44 Hz (Tan et al., 2024), in which case the multiplet appears like a quartet. The ${}^{19}F$ resonances of residue 5 in GB1-1 and GB1-2 are examples of these two limiting cases (Fig. [Fig F4]c and d).

**Figure 4 F4:**
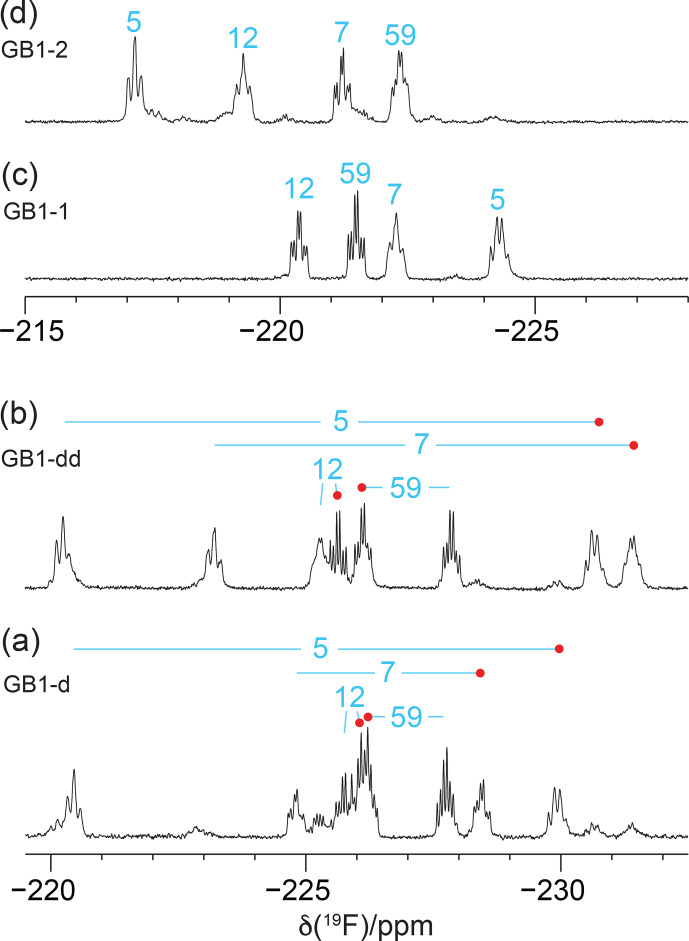
1D ${}^{19}F$ NMR spectra without ${}^{1}H$ decoupling recorded of the samples from Fig. [Fig F3]. **(a)** GB1-d made with diFLeu, **(b)** GB1-dd made with diFLeu diluted with canonical L-leucine, **(c)** GB1-1 made with FLeu1, and **(d)** GB1-2 made with FLeu2. The resonance assignments are indicated in blue. Red dots identify stereospecific assignments of the $C^{\delta 1}H_{2}F$ groups in diFLeu residues.

Interestingly, the multiplet of the $C^{\delta 1}H_{2}F$ group of residue 12 in GB1-dd displays narrower lines than the $C^{\delta 2}H_{2}F$ group (Fig. [Fig F4]b) in agreement with a narrower signal in GB1-1 than in GB1-2 (Fig. [Fig F3]). This observation aligns with the greater solvent exposure of the $C^{\delta 1}H_{2}F$ group (Fig. [Fig F2]). The inverse correlation between ${}^{19}F$ NMR line width and solvent exposure suggests that faster rotation of the ${\mathrm{CH}}_{2}F$ groups about the $C^{\gamma }\text{–}C^{\delta }$ bond results in slower transverse relaxation.

### 1.12 NMR resonance assignments

The large $J_{\mathrm{HF}}$ couplings observed indicate that resonance assignments can be achieved by coherence transfer between ${}^{1}H$ and ${}^{19}F$ spins and linking the ${}^{1}H$ resonances of the isopropyl groups to the backbone protons by [ ${}^{1}H$ , ${}^{1}H$ ]-TOCSY and [ ${}^{1}H$ , ${}^{1}H$ ]-NOESY spectra. The ${}^{1}H$ chemical shifts of the ${\mathrm{CH}}_{2}F$ groups are near 4 ppm , and the methine resonances are between 1 and 2 ppm . For GB1 made with diFLeu, a [ ${}^{1}H$ , ${}^{19}F$ ]-COSY spectrum connected the ${}^{19}F$ NMR signals belonging to the same residue (Fig. [Fig F5]).

**Figure 5 F5:**
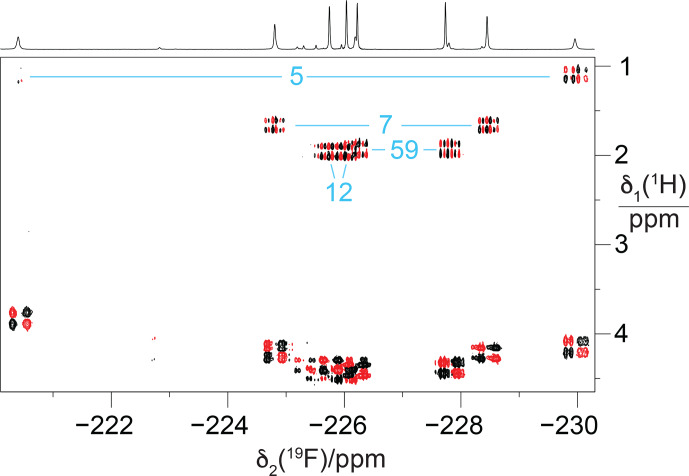
[ ${}^{1}H$ , ${}^{19}F$ ] correlation spectrum of a concentrated solution of GB1-d (about 10 mM ). The ${}^{1}H$ -decoupled 1D ${}^{19}F$ NMR spectrum is shown at the top. The [ ${}^{1}H$ , ${}^{19}F$ ]-COSY spectrum was recorded with the pulse sequence 90°( ${}^{1}H$ ) – $t_{1}$ – 90°( ${}^{1}H$ ),90°( ${}^{19}F$ ) – acquisition( ${}^{19}F$ ). The cross peaks with the methine proton of the isopropyl groups, which identify the pairs of ${\mathrm{CH}}_{2}F$ groups belonging to the same residue, are assigned in blue. Parameters: $t_{1\text{max}}$ = 51 ms , $t_{2\text{max}}$ = 217 ms , 1.4 h total recording time.

To probe for the presence of scalar through-space ${}^{19}F{\text{–}}^{19}F$ couplings in GB1-d, we recorded a [ ${}^{19}F$ , ${}^{19}F$ ]-TOCSY spectrum. The spectrum yielded both intra-residual and through-space correlations (Fig. [Fig F6]a). Interestingly, the intra-residual cross peak of residue 5 could not be observed, whereas the inter-residual connectivities with the nearest neighbour (residue 7) were intense. Residue 7 in turn showed cross peaks to residues 5 and 12, which were more intense than the intra-residual cross peaks. The absence of the intra-residual cross peak of residue 5 indicates that scalar ${}^{4}J_{\mathrm{FF}}$ couplings cannot be relied upon to connect the ${}^{19}F$ NMR signals of the ${\mathrm{CH}}_{2}F$ groups of each diFLeu residue.

**Figure 6 F6:**
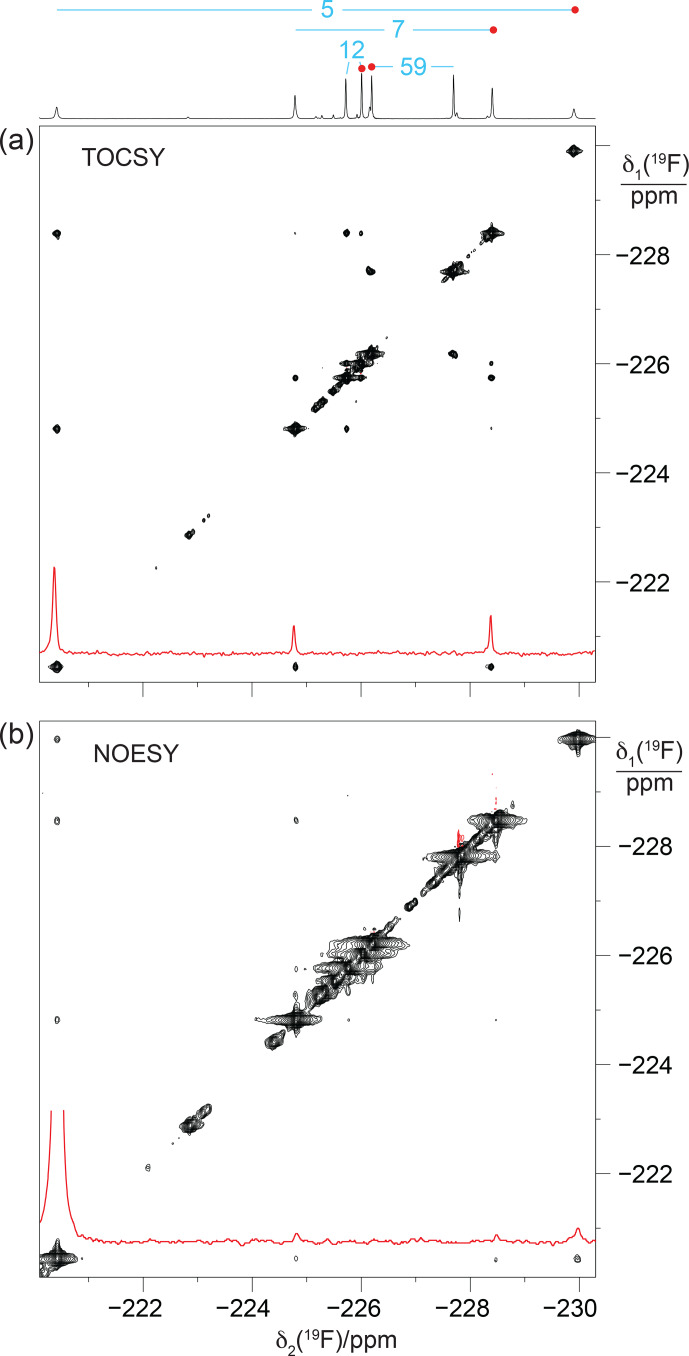
[ ${}^{19}F$ , ${}^{19}F$ ]-TOCSY and [ ${}^{19}F$ , ${}^{19}F$ ]-NOESY spectra of GB1-d. The 1D ${}^{19}F$ NMR spectrum is shown at the top along with the resonance assignments. Cross sections through the diagonal peaks at - 220.4 ppm are shown in red. **(a)** [ ${}^{19}F$ , ${}^{19}F$ ]-TOCSY spectrum (mixing time 60 ms ) recorded of an 0.8 mM protein solution. Parameters: DIPSI-2 mixing with 4200 Hz RF field strength, $t_{1\text{max}}$ = 26 ms , $t_{2\text{max}}$ = 105 ms , 14 h total recording time. **(b)** [ ${}^{19}F$ , ${}^{19}F$ ]-NOESY spectrum (mixing time 200 ms ) recorded of a > 10 mM solution of GB1-d. Parameters: $t_{1\text{max}}$ = 13.5 ms , $t_{2\text{max}}$ = 108 ms , 12 h total recording time, processed with 20 Hz exponential line broadening in the $\delta_{2}$ dimension. Without cropping, the diagonal peak in the cross section would exceed the boundary of panel **(b)** twofold.

Notably, some of the most intense [ ${}^{19}F$ , ${}^{19}F$ ]-TOCSY cross peaks came about by ${}^{\mathrm{TS}}J_{\mathrm{FF}}$ couplings. To exclude the possibility of TOCSY cross peaks arising from ${}^{19}F{\text{–}}^{19}F$ nuclear Overhauser effects (NOEs), we also recorded a [ ${}^{19}F$ , ${}^{19}F$ ]-NOESY spectrum (Fig. [Fig F6]b). The NOESY spectrum produced the intra-residual cross peak of residue 5 with greater intensity than the inter-residual NOEs. This illustrates the different dependence of NOEs and ${}^{\mathrm{TS}}J_{\mathrm{FF}}$ couplings on the internuclear distance, with ${}^{\mathrm{TS}}J_{\mathrm{FF}}$ couplings depending on close contacts between the fluorine atoms to create the necessary orbital overlap. Notably, although the NOESY spectrum had been recorded of a GB1-d sample with over 10-fold-higher protein concentration, the cross-peak intensities were markedly poorer in the NOESY than in the TOCSY spectrum.

The GB1 samples prepared with FLeu1 or FLeu2 (GB1-1 and GB1-2, respectively) offer fewer opportunities for ${}^{\mathrm{TS}}J_{\mathrm{FF}}$ couplings. The conformation shown in Fig. [Fig F2] excludes direct contacts between $C^{\delta 1}H_{3}$ groups, whereas van der Waals contacts between ${}^{19}F$ atoms of $C^{\delta 2}H_{2}F$ groups are arguably possible in view of the greater C–F bond length and larger van der Waals radius of fluorine compared with hydrogen. Even so, direct fluorine–fluorine contacts in GB1-2 depend on specific rotamer combinations of neighbouring ${\mathrm{CH}}_{2}F$ groups and may be infrequent if the ${\mathrm{CH}}_{2}F$ groups rotate.

Experimentally, residue 7 produced inter-residual cross peaks in the [ ${}^{19}F$ , ${}^{19}F$ ]-TOCSY spectra of GB1-1 and GB1-2 (Fig. [Fig F7]). In the case of GB1-1, the cross peaks were about 100 times smaller than the diagonal peaks. In the case of GB1-2, residue 7 produced cross peaks both with residue 5 and residue 12, and those cross peaks were only about 10 times smaller than the diagonal peaks. No single conformation of the $C^{\delta 2}H_{2}F$ group of residue 7 can simultaneously engage in fluorine–fluorine contacts with residues 5 and 12 (Fig. [Fig F2]), suggesting that the $C^{\delta 2}H_{2}F$ group populates multiple rotamers. Furthermore, the cross peak observed between the $C^{\delta 1}H_{2}F$ groups of residues 7 and 12 in GB1-1 suggests that these side chains enjoy greater conformational freedom than captured by the NMR structure 3GB1, which was determined with the aim of presenting the single best approximation to the average structure.

**Figure 7 F7:**
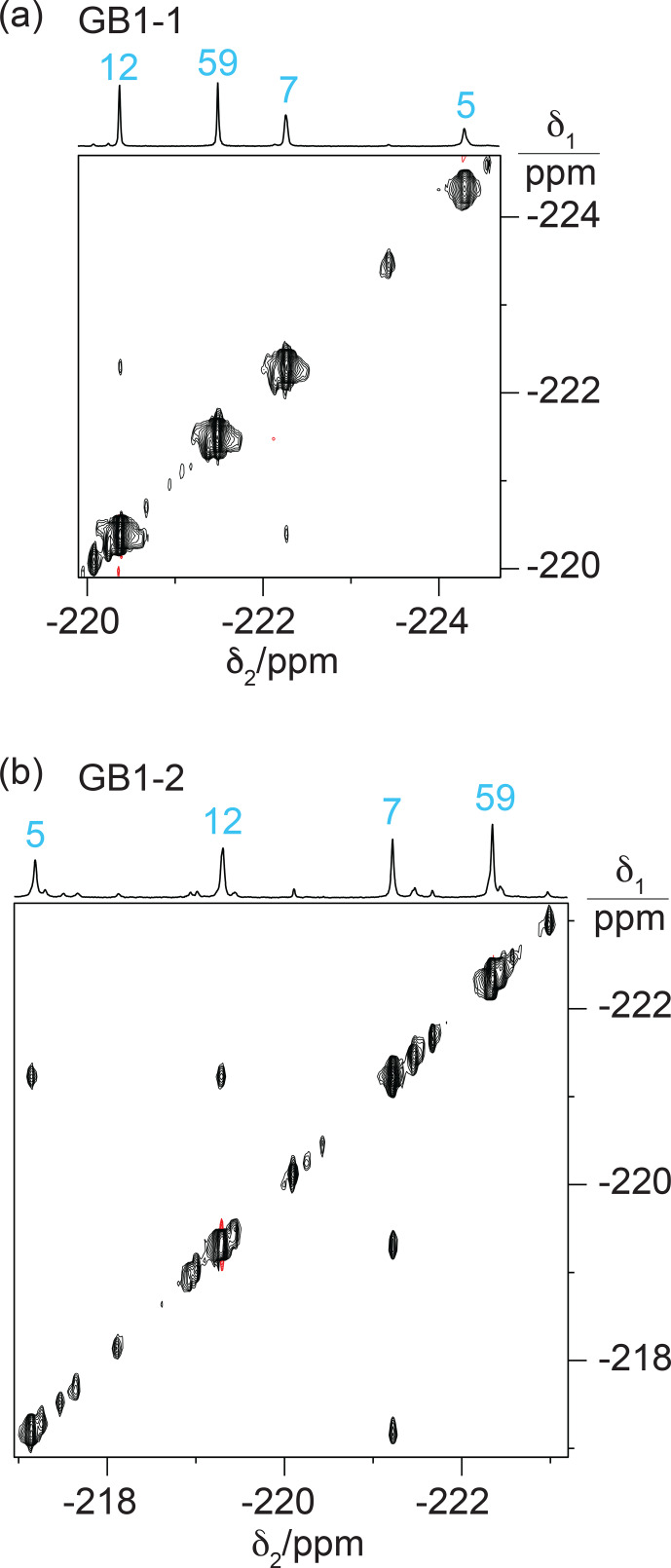
[ ${}^{19}F$ , ${}^{19}F$ ]-TOCSY spectra of 2 mM solutions of GB1-1 and GB1-2 recorded with 60 ms mixing time. The 1D NMR spectra are plotted on top with the resonance assignments in blue. **(a)** TOCSY spectrum of GB1-1 recorded in about 3 h using $t_{1\text{max}}$ = 8.5 ms and $t_{2\text{max}}$ = 128 ms . The cross peaks between residues 7 and 12 are about 100 times weaker than the diagonal peaks. **(b)** TOCSY spectrum of GB1-2 recorded in about 12 h using $t_{1\text{max}}$ = 8 ms and $t_{2\text{max}}$ = 171 ms . The cross peaks are about 10 times smaller than the diagonal peaks.

The fluorine–fluorine contacts observed in GB1-2 recapitulate the two strongest cross peaks observed with the high-field ${}^{19}F$ resonance of residue 7 in GB1-d (Fig. [Fig F6]a). Assuming that the side-chain conformations are conserved between GB1-2 and GB1-d, this affords stereospecific assignments of GB1-d, assigning the high-field signals of residues 7 and 12 and the low-field signal of residue 5 to the ${}^{19}F$ spins of the respective $C^{\delta 2}H_{2}F$ groups. Given this assignment, the weaker interaction between the low-field signals of residues 7 and 12 indicates a contact between a $C^{\delta 1}H_{2}F$ and a $C^{\delta 2}H_{2}F$ group, which cannot occur in either GB1-1 or GB1-2. The generally greater cross-peak intensities observed in GB1-d may be a consequence of the greater steric crowding associated with the spatial demands of multiple fluorine atoms, bringing the ${}^{19}F$ spins into closer contact. In addition, the greater density of ${}^{19}F$ spins in GB1-d opens the chance for multiple magnetisation transfer steps during the TOCSY mixing period.

### 1.13 Estimates of ${}^{\mathrm{TS}}J_{\mathrm{FF}}$ from [ ${}^{19}F$ , ${}^{19}F$ ]-TOCSY spectra

Using the sample of GB1-d produced with deuterated diFLeu (Fig. S4a in the Supplement) to minimise the relaxation of ${}^{19}F$ , we recorded [ ${}^{19}F$ , ${}^{19}F$ ]-TOCSY spectra with increasing mixing time $\tau_{m}$ (27.6, 41.4, and 55.3 ms ), measured the integrals of cross peaks ( $I_{C}$ ) and diagonal peaks ( $I_{D}$ ) and calculated the ratio $I_{C}/I_{D}$ . In the approximation of a two-spin system and assuming that the cross peaks and diagonal peaks relax at the same rate, the $I_{C}/I_{D}$ ratio is expected to evolve during isotropic mixing with ${\text{tan}}^{2}(\pi J_{\mathrm{FF}}\tau_{m}$ ) (Braunschweiler and Ernst, 1983). The largest $J_{\mathrm{FF}}$ couplings found in this way were about 2–3 Hz (Fig. S5 in the Supplement).

### 1.14 Heteronuclear NMR for residue assignment

Heteronuclear [ ${}^{1}H$ , ${}^{19}F$ ]-NOESY (HOESY) spectra recorded with 150 ms mixing times showed NOEs with nearby protons (Fig. [Fig F8]). These NOEs delivered residue-specific resonance assignments as many of the corresponding ${}^{1}H$ nuclei were also detected in conventional homonuclear [ ${}^{1}H$ , ${}^{1}H$ ]-NOESY spectra. For example, residue 5 in GB1-1 and GB1-d displays NOEs to a ${}^{1}H$ resonance at about - 0.8 ppm . This resonance matches a β -proton of Leu5, which in wild-type GB1 is the most high-field ${}^{1}H$ resonance due to aromatic ring currents from Phe28. In all three samples, the ${}^{19}F$ NMR signal of residue 5 produced stronger HOESY cross peaks than the other fluorinated leucine residues, while residue 59 delivered relatively weak cross peaks if any. This result indicates that a ${\mathrm{CH}}_{2}F$ group produces stronger HOESY cross peaks when it is buried in the core of the protein than when it is solvent exposed and can rotate in an unhindered manner. The ${}^{19}F$ NMR assignments of residue 7 were confirmed similarly by comparison of the cross peaks observed in the HOESY and [ ${}^{1}H$ , ${}^{1}H$ ]-NOESY spectra.

**Figure 8 F8:**
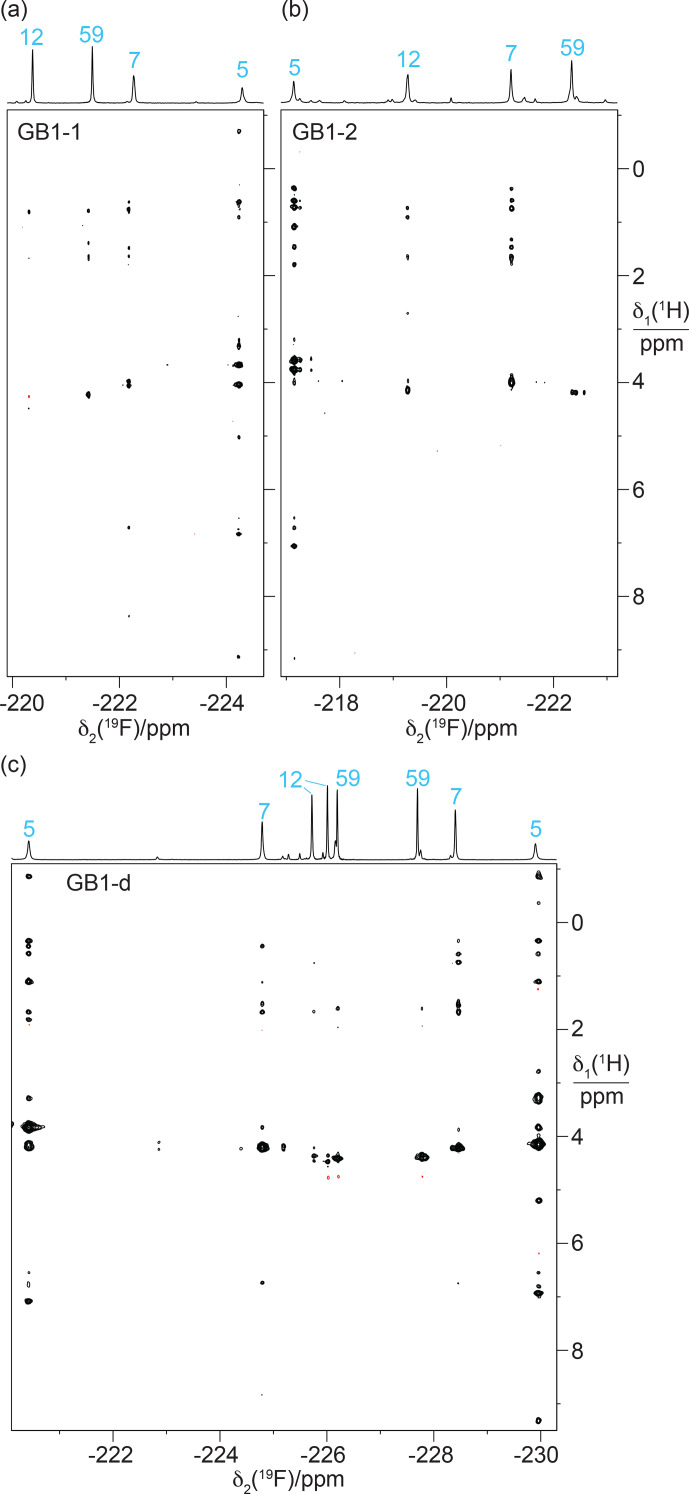
[ ${}^{1}H$ , ${}^{19}F$ ]-HOESY spectra of GB1 produced with FLeu1, FLeu2, or diFLeu. The spectra were recorded with a mixing time of 150 ms . The corresponding 1D ${}^{19}F$ NMR spectra are shown at the top along with the resonance assignments. **(a)** HOESY spectrum of a 2.2 mM solution of GB1-1 in MES buffer, recorded using $t_{1\text{max}}$ = 38 ms , $t_{2\text{max}}$ = 136 ms , 9.6 h total recording time. **(b)** HOESY spectrum of a 2 mM solution of GB1-2, recorded using $t_{1\text{max}}$ = 30 ms , $t_{2\text{max}}$ = 136 ms , 34 h total recording time. **(c)** HOESY spectrum of a 10 mM solution of GB1-d, recorded using $t_{1\text{max}}$ = 31 ms , $t_{2\text{max}}$ = 108 ms , 2.3 h total recording time.

In the case of the diFLeu residue in position 12, stronger cross peaks were detected for the low-field signal assigned to the $C^{\delta 2}H_{2}F$ than the $C^{\delta 1}H_{2}F$ group (Fig. [Fig F8]c). In addition, the $C^{\delta 1}H_{2}F$ group of this residue displays a negative cross peak with the water resonance (at 4.75 ppm ), as do both ${}^{19}F$ NMR signals of residue 59, indicating intermolecular NOEs with hydration water molecules featuring sub-nanosecond residence times (Otting et al., 1991). This confirms the solvent exposure of these fluorine atoms and agrees with the stereospecific assignments of residue 12 made by comparing the ${}^{\mathrm{TS}}J_{\mathrm{FF}}$ couplings with the protein structure. We observed no other negative NOE cross peaks with the ${\mathrm{CH}}_{2}F$ groups.

Starting from the assignment of residue 5, the cross peaks observed in the [ ${}^{19}F$ , ${}^{19}F$ ]-TOCSY spectra provided an additional, straightforward assignment pathway for the ${}^{19}F$ spins in GB1-d and GB1-2 (Fig. [Fig F6]b and 7b). In GB1-dd, as in GB1-1, the $C^{\delta 1}H_{2}F$ group of residue 12 produced only weak HOESY cross peaks. The HOESY spectrum thus did not identify the ${}^{19}F$ NMR signals belonging to the same diFLeu residue in position 12. This link, however, was easily established by correlations with the γ proton of the isopropyl group observed in a short-delay ${}^{1}H$ , ${}^{19}F$ correlation experiment.

### 1.15 Measurement of ${}^{3}J_{\mathrm{HF}}$ couplings and γ -gauche effect

${}^{3}J_{\mathrm{HF}}$ couplings are governed by a Karplus relationship describing their dihedral angle dependence, and thus provide information about the rotameric states of the ${\mathrm{CH}}_{2}F$ groups. Quantitative measurements of J couplings in the 1D ${}^{19}F$ NMR spectra recorded without ${}^{1}H$ decoupling were hampered by spectral overlap and the presence of sample heterogeneities (Fig. [Fig F3]). Narrower ${}^{19}F$ multiplets were obtained for samples prepared with diFLeu versions that had been synthesised with ${\mathrm{CD}}_{2}F$ instead of ${\mathrm{CH}}_{2}F$ groups (Maleckis et al., 2022), where ${}^{3}J_{\mathrm{HF}}$ couplings were manifested in a sample made with diFLeu containing a $C^{\gamma }H$ group, whereas these splittings were absent from a sample prepared with deuterated diFLeu containing a $C^{\gamma }D$ group. A simple comparison of these spectra shows a correlation between the ${}^{19}F$ chemical shifts and splittings due to ${}^{3}J_{\mathrm{HF}}$ couplings (Fig. S4).

For more quantitative measurements of the ${}^{3}J_{\mathrm{HF}}$ couplings, we recorded short-delay ${}^{1}H$ , ${}^{19}F$ correlation experiments (Tan et al., 2024), which encode the ${}^{3}J_{\mathrm{HF}}$ coupling constants in the relative peak intensities of $H^{\gamma }{\text{–}}^{19}F$ versus $H^{\delta }{\text{–}}^{19}F$ cross peaks. The results confirm the correlation between the ${}^{3}J_{\mathrm{HF}}$ coupling constants and the ${}^{19}F$ chemical shifts (Fig. [Fig F9]). This correlation is a hallmark of the γ -gauche effect, which associates a high-field ${}^{19}F$ chemical shift with the rotameric state of the ${\mathrm{CH}}_{2}F$ group that positions the ${}^{19}F$ spin *trans* relative to the γ proton of the isopropyl group (Feeney et al., 1996; Tan et al., 2024; Frkic et al., 2024a, b). Conversely, the ${}^{19}F$ NMR resonance is shifted low-field if the ${}^{19}F$ spin is positioned *trans* relative to a carbon atom. The γ -gauche effect is most clearly illustrated by the low-field and high-field signals of residue 5.

**Figure 9 F9:**
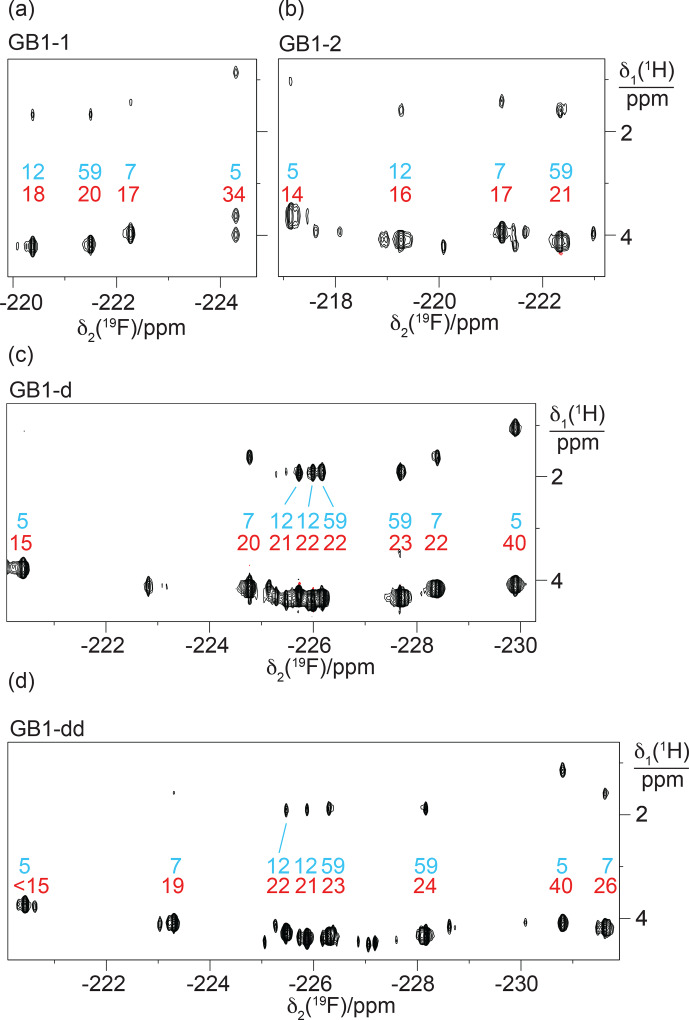
Short-delay ${}^{1}H$ , ${}^{19}F$ correlation experiments for the measurement of ${}^{3}J_{\mathrm{HF}}$ coupling constants. The experiments were conducted with a ${}^{1}H$ constant-time evolution period of 7 ms to evolve the $J_{\mathrm{HF}}$ couplings and a refocusing INEPT period of 2.5 ms (Tan et al., 2024). The resonance assignments are indicated in blue. Red numbers indicate the ${}^{3}J_{\mathrm{HF}}$ coupling constants (in Hz) derived from the relative intensities of the $H^{\gamma }$ – ${}^{19}F$ versus $H^{\delta }$ – ${}^{19}F$ cross peaks. **(a)** Spectrum recorded of a 2 mM solution of GB1-1. Parameters: $t_{1\text{max}}$ = 7 ms , $t_{2\text{max}}$ = 128 ms , 1.3 h total recording time. **(b)** Spectrum recorded of a 2 mM solution of GB1-2 using $t_{1\text{max}}$ = 6.9 ms , $t_{2\text{max}}$ = 171 ms , 2.8 h total recording time. **(c)** Spectrum recorded of a 0.8 mM solution of GB1-d using $t_{1\text{max}}$ = 7 ms , $t_{2\text{max}}$ = 180 ms , 5.3 h total recording time. **(d)** Spectrum recorded of a 0.6 mM solution of GB1-dd produced with diFLeu diluted with Leu using $t_{1\text{max}}$ = 7 ms , $t_{2\text{max}}$ = 181 ms , 22.3 h total recording time.

The immediate chemical environment of the ${}^{19}F$ spins also affects their chemical shifts. For example, the ${}^{3}J_{\mathrm{HF}}$ coupling of the high-field ${}^{19}F$ resonance of residue 7 in GB1-dd is smaller than for residue 5 (26 Hz versus 40 Hz ; Table S4 in the Supplement), yet the resonance appears more high-field in the spectrum (Fig. [Fig F9]d). Quite generally, the ${}^{19}F$ chemical shifts of residue 7 are very sensitive to the presence or absence of fluorinated residues in positions 5 and 12 (Fig. [Fig F9]c and d), highlighting the impact of the chemical environment.

Interestingly, the ${}^{19}F$ spins of residue 7 showed significantly different ${}^{3}J_{\mathrm{HF}}$ couplings between the GB1-d and GB1-dd preparations, suggesting somewhat different populations of the different rotameric states of the ${\mathrm{CH}}_{2}F$ groups. The associated changes in ${}^{19}F$ chemical shifts are high-field and low-field as predicted by the γ -gauche effect.

Very large and very small ${}^{3}J_{\mathrm{HF}}$ couplings indicate that ${\mathrm{CH}}_{2}F$ groups are trapped in pure *trans* or *gauche* rotamers, respectively, showing that the rotation of a ${\mathrm{CH}}_{2}F$ group about the $C^{\gamma }\text{–}C^{\delta }$ bond axis can be halted by the steric restraints in the tightly packed core of the protein. In the case of the *E. coli* peptidyl–prolyl *cis*–*trans* isomerase B (PpiB) produced with FLeu and diFLeu, we determined ${}^{3}J_{\mathrm{HF}}$ couplings ranging between 9 and 44 Hz (Tan et al., 2024). The ${}^{3}J_{\mathrm{HF}}$ couplings observed in GB1 are less extreme, suggesting that each ${\mathrm{CH}}_{2}F$ group populates more than a single rotamer. Using residue 59 located in the flexible TEV cleavage motif as a reference, a ${}^{3}J_{\mathrm{HF}}$ coupling of about 22 Hz is indicative of a ${\mathrm{CH}}_{2}F$ group that populates all three possible staggered rotamers. The different ${}^{3}J_{\mathrm{HF}}$ couplings observed for residue 5 (Table S4), which is the most deeply buried leucine side chain in the wild-type protein, thus indicate clear conformational preferences for its ${\mathrm{CH}}_{2}F$ groups. The largest ${}^{3}J_{\mathrm{HF}}$ couplings were observed for diFLeu rather than FLeu1 or FLeu2 residues, as expected for fluorine–fluorine interactions biasing the conformational space of the ${\mathrm{CH}}_{2}F$ groups (Marstokk and Møllendal, 1997; Wu et al., 1998; Lu et al., 2019).

On a technical note, the short-delay ${}^{1}H$ , ${}^{19}F$ correlation experiments delivered the $H^{\gamma }$ chemical shifts with much greater sensitivity than the [ ${}^{1}H$ , ${}^{19}F$ ]-COSY experiment recorded without heteronuclear decoupling (Fig. [Fig F5]), assisting with the resonance assignments by comparison with [ ${}^{1}H$ , ${}^{1}H$ ]-NOESY spectra. In terms of sensitivity, the short-delay ${}^{1}H$ , ${}^{19}F$ correlation experiments were also far superior to the HOESY spectra. The chemical shifts of the $H^{\gamma }$ spins were well conserved between the samples made with FLeu1, FLeu2, and diFLeu, ascertaining the ${}^{19}F$ resonance assignment of GB1-dd by comparison with GB1-d.

### 1.16 ${}^{13}C$ -NMR spectroscopy

The ${}^{13}C$ chemical shifts of the ${\mathrm{CH}}_{3}$ groups in the singly fluorinated leucine analogues FLeu1 and FLeu2 were shifted upfield by between 5.6 and 8.2 ppm relative to the shifts of the methyl groups in the wild-type protein (Fig. S6 in the Supplement). Highly conserved ${}^{1}H$ and ${}^{13}C$ chemical shifts of the GB1 variants indicate that the 3D fold of the protein remains unchanged by the fluorinated leucine analogues. Therefore, any differences in chemical shifts reflect local rather than global effects. The ${}^{13}C$ heteronuclear single-quantum coherence ( ${}^{13}C$ -HSQC) spectra showed the cross peaks of the ${\mathrm{CH}}_{2}F$ groups in the ${}^{13}C$ dimension near 90 ppm for GB1-1 and GB1-2 and about 86 ppm for GB1-d (Fig. S7). In the ${}^{1}H$ dimension, most ${\mathrm{CH}}_{2}F$ groups displayed two different chemical shifts for the diastereotopic ${}^{1}H$ spins, which, except for residue 5 in GB1-1, were unresolved in the short-delay ${}^{1}H$ , ${}^{19}F$ correlation experiments (Fig. [Fig F9]). The intensities of the ${}^{13}C$ -HSQC cross peaks of the ${\mathrm{CH}}_{2}F$ groups of residue 5 were rather weak (similar to those of ${\mathrm{CH}}_{2}$ groups of other buried amino acid residues), which correlates with the relatively broad ${}^{19}F$ NMR signals observed for this residue. The other ${\mathrm{CH}}_{2}F$ groups showed more intense ${}^{13}C$ -HSQC cross peaks on par with solvent-exposed ${\mathrm{CH}}_{2}$ groups. The methyl cross peaks of Leu5 are relatively weak also in wild-type GB1 (Fig. S6; Goehlert et al., 2004).

## 2 Discussion

The conformational impact of the fluorination of leucine methyl groups has previously been investigated in solution for only a single protein, PpiB, which contains five isolated leucine residues (Tan et al., 2024; Frkic et al., 2024a). The current findings recapitulate many of the findings made for PpiB.

i. In the case of the most buried residue, residue 5, the rotation of the ${\mathrm{CH}}_{2}F$ groups is sufficiently hindered to bias the populations of the different rotamers in favour of a *trans* configuration of the $C^{\delta 1}H_{2}F$ group and a *gauche* configuration of the $C^{\delta 2}H_{2}F$ group as defined by the ${}^{3}J_{\mathrm{HF}}$ coupling constants. The size of the ${}^{3}J_{\mathrm{HF}}$ couplings indicates that these conformational biases are more pronounced in GB1-d than in GB1-1 and GB1-2, which may be attributed to unfavourable electrostatic interactions between parallel and antiparallel C–F bonds in a 1,3-difluoropropane moiety intrinsically limiting the conformational freedom (Marstokk and Møllendal, 1997; Wu et al., 1998; Lu et al., 2019).
ii. The large chemical shift dispersion of the ${}^{19}F$ NMR signals over many parts per million is mainly due to the γ -gauche effect, which attributes high-field and low-field shifts to *trans* and *gauche* rotamers (Feeney et al., 1996). Intermediate chemical shifts correlate with intermediate ${}^{3}J_{\mathrm{HF}}$ coupling constants and are thus indicative of averaging between different rotamers. A γ -gauche effect in leucine side chains has previously been reported also for the ${}^{13}C$ chemical shifts of leucine $C^{\delta }H_{3}$ groups, which correlate with ${}^{3}J_{\mathrm{CC}}$ couplings with the α -carbon (MacKenzie et al., 1996). To the best of our knowledge, the present work is only the third experimental example of the γ -gauche effect in ${\mathrm{CH}}_{2}F$ groups (Fig. S8; Tan et al., 2024; Frkic et al., 2024b).
iii. The more solvent accessible ${\mathrm{CH}}_{2}F$ groups displayed less extreme ${}^{3}J_{\mathrm{HF}}$ couplings and less extreme ${}^{19}F$ chemical shifts, suggesting more extensive averaging between different rotameric states. Larger and smaller ${}^{3}J_{\mathrm{HF}}$ couplings as well as greater ${}^{19}F$ chemical shift dispersions have been observed previously in PpiB (Tan et al., 2024), suggesting that the ${\mathrm{CH}}_{2}F$ groups populate more than a single rotamer even in the buried residue 5.
iv. The line widths of the ${}^{19}F$ NMR signals vary greatly between different residues and, most strikingly for residue 12, between different ${\mathrm{CH}}_{2}F$ groups. Narrow signals correlate with high solvent exposure. For wild-type GB1, order parameter $S_{axis}^{2}$ values determined by relaxation measurements have been reported for the methyl groups of Leu12 ( < 0.15), Leu7 $C^{\delta 2}H_{3}$ (0.15), and Leu5 $C^{\delta 1}H_{3}$ (0.55), showing that the methyl group symmetry axes are subject to motions, which are more prominent in situations of high solvent exposure (Goehlert et al., 2004). The relatively high order parameter of Leu5 correlates with ${}^{19}F$ NMR signals that are broader than any others.
v. For any given position in the protein, the relative chemical shifts observed between FLeu1 and FLeu2 are strongly predictive of the stereospecific assignments of a diFLeu residue at the same site. The same feature also prevails in PpiB (Tan et al., 2024).

The present work shows, for the first time, that through-space ${}^{19}F{\text{–}}^{19}F$ couplings can readily be detected between singly fluorinated ${\mathrm{CH}}_{2}F$ groups in a protein. In previous work, we detected ${}^{\mathrm{TS}}J_{\mathrm{FF}}$ couplings between genetically encoded ${\mathrm{CF}}_{3}$ -phenylalanine and ${\mathrm{CF}}_{3}$ -tyrosine residues in the core of PpiB (Orton et al., 2021). Notably, however, the ${}^{19}F$ NMR spectra of PpiB constructs with multiple ${\mathrm{CF}}_{3}$ groups showed additional resonances, suggesting structural perturbations arising from the additional space requirements of ${\mathrm{CF}}_{3}$ groups. The ${}^{19}F$ NMR spectra of GB1 made with fluorinated leucine analogues also display weak additional resonances, but there is no evidence that they are due to structural heterogeneity. Instead, the additional signals are consistent with chemical heterogeneity arising from incomplete substitution of canonical leucine by fluorinated leucines or incomplete optical purity of the synthesised fluoroleucine. Cell-free protein synthesis enables the requisite high level of global substitution of canonical amino acids by fluorinated analogues.

The observation of ${}^{\mathrm{TS}}J_{\mathrm{FF}}$ couplings in GB1 is non-trivial as they depend on direct contact between the fluorine atoms. Crystal structures of PpiB showed that ${\mathrm{CH}}_{2}F$ groups often populate multiple staggered rotamers that differ by rotation about the bond with the carbon atom they are bound to (Frkic et al., 2024a, b). Based on the 3D structure of wild-type GB1 (Fig. [Fig F2]), only specific rotamer combinations generate fluorine–fluorine contacts. A crystal structure of ubiquitin synthesised chemically with two FLeu1 residues indicated that the lowest energy conformation avoids fluorine–fluorine contacts (Alexeev et al., 2003). In the case of 1,3-difluoropropane, it is known that the polarity of C–F bonds discourages rotamers that produce fluorine–fluorine contacts (Marstokk and Møllendal, 1997; Wu et al., 1998; Lu et al., 2019). Therefore, the privileged attraction between fluorine atoms in perfluorinated polymers such as Teflon does not govern the interaction between the single fluorine atoms of ${\mathrm{CH}}_{2}F$ groups. Nonetheless, the transient ${}^{19}F{\text{–}}^{19}F$ contacts arising from random rotations of the ${\mathrm{CH}}_{2}F$ groups in GB1-1, GB1-2, and GB1-d suffice to generate observable ${}^{\mathrm{TS}}J_{\mathrm{FF}}$ couplings. As noted previously (Orton et al., 2021; Tan et al., 2024), the much steeper distance dependence of ${}^{\mathrm{TS}}J_{\mathrm{FF}}$ couplings compared with ${}^{19}F{\text{–}}^{19}F$ NOEs (Ernst and Ibrom, 1995; Mallory et al., 2000) strongly favours the detection of transient fluorine–fluorine contacts by [ ${}^{19}F$ , ${}^{19}F$ ]-TOCSY rather than [ ${}^{19}F$ , ${}^{19}F$ ]-NOESY experiments (Fig. [Fig F6]).

For the side chain of Leu12 in GB1, a very different $\chi_{2}$ angle has been reported by the crystal structures (1PGA, 1PGB; Gallagher et al., 1994; 2QMT; Frericks Schmidt et al., 2007) versus the NMR solution structure (3GB1; Kuszewski et al., 1999). As a result, the crystal structures expose the $\delta_{2}$ methyl group to the solvent, while the solution structure exposes the $\delta_{1}$ methyl group. The observation of ${}^{1}H{\text{–}}^{19}F$ NOEs with water together with different ${}^{19}F$ NMR line widths indicative of more facile rotation of the $C^{\delta 1}H_{2}F$ than $C^{\delta 2}H_{2}F$ group fully agree with the conformation of Leu12 depicted in Fig. [Fig F2], indicating that fluorinated leucine residues do not alter the solution structure. Simple rotations of the ${\mathrm{CH}}_{2}F$ groups allow for accommodating the fluorine atoms in the energetically most favourable rotamers.

Establishing sequence-specific resonance assignments of the ${}^{19}F$ NMR spectra by 2D NMR techniques rather than site-directed mutagenesis is straightforward for small proteins like GB1. For larger proteins, site-specific selective installation of the fluorinated amino acids by genetic encoding (Orton et al., 2021; Qianzhu et al., 2020, 2022, 2024) will be helpful. Work towards this goal is in progress.

## Supplement

10.5194/mr-6-131-2025-supplement The supplement related to this article is available online at https://doi.org/10.5194/mr-6-131-2025-supplement.

## Supplement

10.5194/mr-6-131-2025-supplement
10.5194/mr-6-131-2025-supplement The supplement related to this article is available online at https://doi.org/10.5194/mr-6-131-2025-supplement.

## Data Availability

The NMR data are available at 10.5281/zenodo.15266133 (Otting, 2025).

## Appendix

The supplement related to this article is available online at https://doi.org/10.5194/mr-6-131-2025-supplement.
