# Supplementary material for: Inter-residue through-space scalar 19F–19F couplings between CH2F groups in a protein
Source: Magn Reson (Gott). 2025 Jul 14;6(2):131–42. doi: 10.5194/mr-6-131-2025 (PMC12281167; doi:10.5194/mr-6-131-2025)
Supplement: The supplement related to this article is available online at https://doi.org/10.5194/mr-6-131-2025-supplement. [file mr-6-131-2025-supplement.pdf]

Supplement of Magn. Reson., 6, 131–142, 2025
https://doi.org/10.5194/mr-6-131-2025-supplement
© Author(s) 2025. CC BY 4.0 License.

Open Access
MAGNETIC
RESONANCE

Supplement of

Inter-residue through-space scalar ^{19}F–^{19}F couplings between CH_2F groups in a protein

Yi Jiun Tan et al.

Correspondence to: Gottfried Otting (gottfried.otting@anu.edu.au)

The copyright of individual parts of the supplement might differ from the article licence.

Supporting Information

Table of contents

Figure S1. Intact protein mass spectra of GB1 made with FLeu1, FLeu2 and diFLeu

Figure S2. Intact protein mass spectra of GB1 made with mixtures of diFLeu and canonical Leu

Figure S3. Thermal stability analysis by circular dichroism (CD) spectroscopy

Figure S4. 1D ^{19}F-NMR spectra of GB1 made with deuterated diFLeu

Figure S5. Determination of $^{TS}J_{FF}$ couplings by [^{19}F,^{19}F]-TOCSY spectra recorded with different mixing times

Figure S6. Methyl region of ^{13}C-HSQC spectra of GB1-1, GB1-2 and wild-type GB1

Figure S7. ^{13}C-HSQC cross-peaks of $C^{\delta}H_2F$ groups in ^{13}C-HSQC spectra of GB1-1, GB1-2 and GB1-d

Figure S8. Correlation between $^{3}J_{HF}$ couplings and ^{19}F chemical shifts, illustrating the γ-gauche effect

Table S1. DNA and corresponding amino acid sequence of the GB1 construct used in the current work

Table S2. Yields of purified GB1 per mL inner CFPS reaction mixture obtained with fluorinated leucine analogues

Table S3. $R_{1\rho}$(^{19}F) values measured in CPMG experiments with different spacing between 180° pulses and comparison with R_2* values

Table S4. Measurements of $^{3}J_{HF}$ couplings by short-delay ^{1}H,^{19}F correlation experiments

References

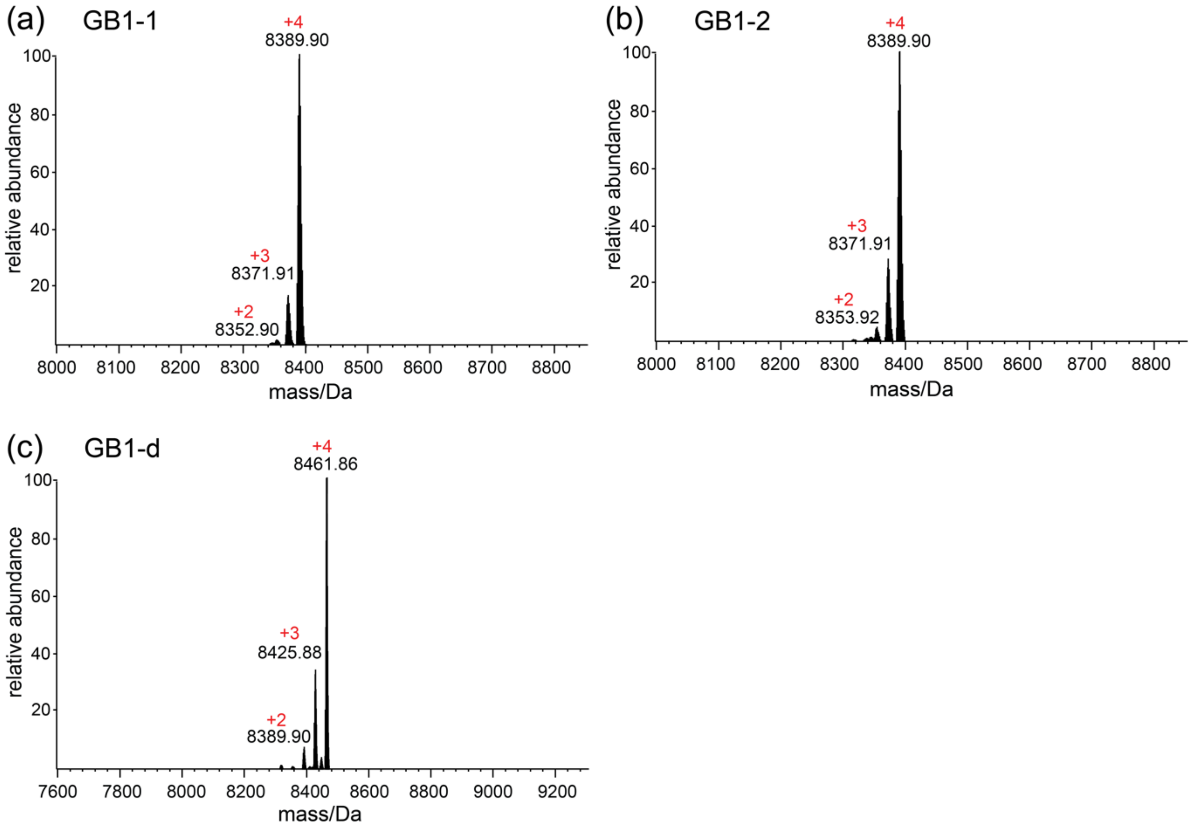

Figure S1. Intact protein mass spectra of GB1 made with (a) Fleu1, (b) FLeu2 and (c) diFLeu. The red numbers indicate the number of FLeu residues in the protein. The expected mass of the full-length protein with four fluorinated leucine residues is 8,390.1 Da for the samples made with FLeu1 and FLeu2, and 8,462.1 Da for the sample made with diFLeu.

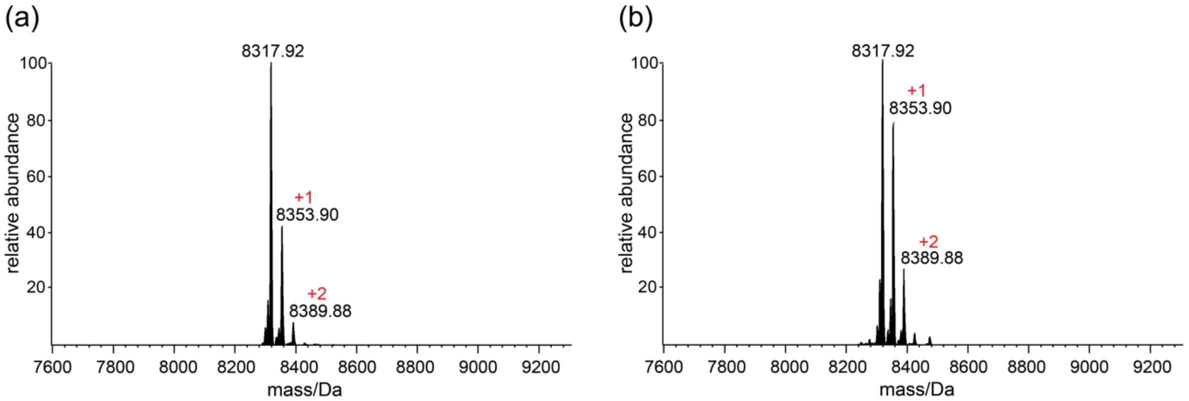

Figure S2. Intact protein mass spectra of GB1 made with mixtures of diFLeu and canonical Leu. (a) Sample prepared with 0.5 mM L-leucine and 4 mM diFLeu in the amino acid mixture of the CFPS reaction. (b) Same as (a) but using 0.25 mM L-leucine and 4 mM diFLeu. The red numbers indicate the number of diFLeu residues in the protein. The expected mass of GB1 containing one or two diFLeu residues is 8,354.1 Da and 8,390.1 Da, respectively.

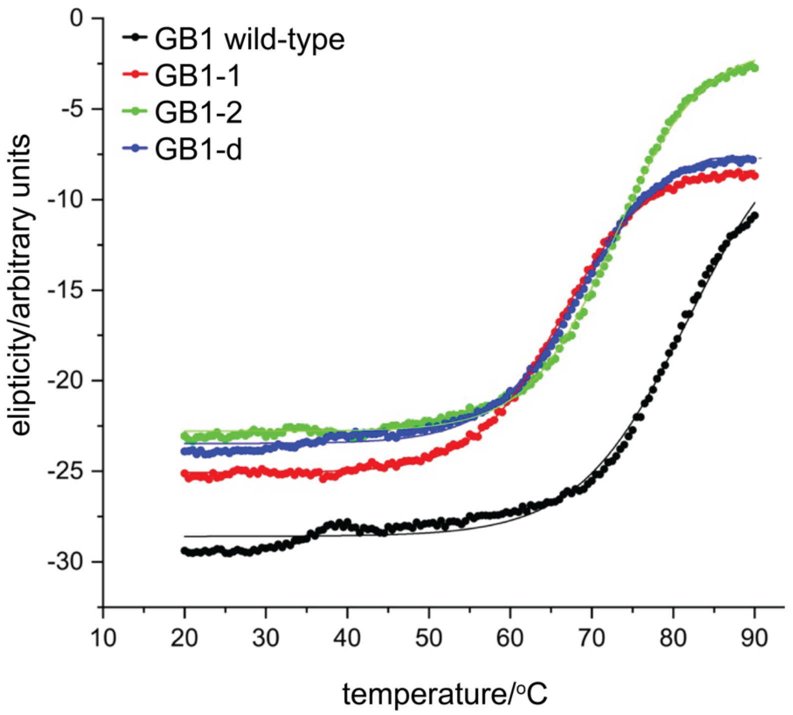

Figure S3. Thermal stability analysis by circular dichroism (CD) spectroscopy. The CD signal of wild-type GB1 (black) and GB1 with FLeu-1 (red), FLeu-2 (green) and diFLeu (blue) was monitored at 216 nm in a 1 mm quartz cuvette using a Chirascan spectrometer (Applied Photophysics). Parameters used: protein concentration about 0.3 mg mL^{-1}, pH 7.5, heating rate 1 °C min^{-1}. Blanks with buffer measured in the same cuvette were subtracted from the data. The melting temperatures obtained from the fits are 81 °C for wild-type GB1, 66 °C for GB1 made with FLeu1 (GB1-1), 72 °C for GB1 made with FLeu2 (GB1-2) and 68 °C for GB1 made with diFLeu (GB1-d).

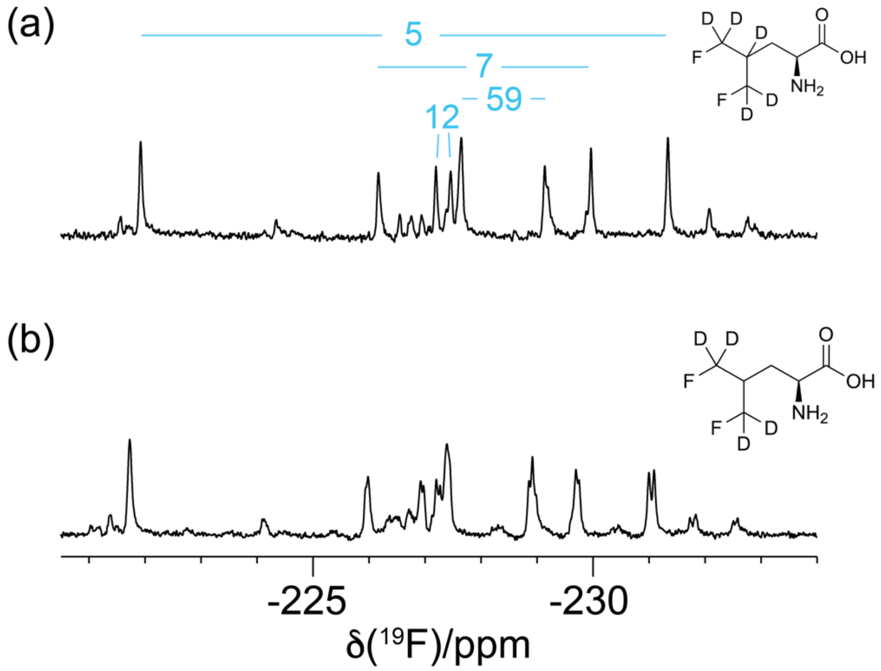

Figure S4. 1D ^{19}F-NMR spectra of GB1 made with diFLeu deuterated at (a) five and (b) four sites as indicated in the chemical structures of the inserts. The spectra were recorded of, respectively, 0.5 and 0.9 mM protein solutions in 20 mM MES buffer pH 6.5, 100 mM NaCl. The spectra were recorded without ^{2}H decoupling, using an acquisition time of 0.1 s and a recovery delay of 1 s. The resonance assignments are shown in cyan. Apart from the deuterium isotope effects on the ^{19}F chemical shifts, the spectra differ by the appearance of $^{3}J_{HF}$ couplings. The spectrum in (b) shows larger splittings at the high-field end of the spectrum while the couplings are unresolved for the low-field resonances, in agreement with the γ-gauche effect that predicts a correlation between ^{19}F chemical shifts and $^{3}J_{HF}$ coupling constants.

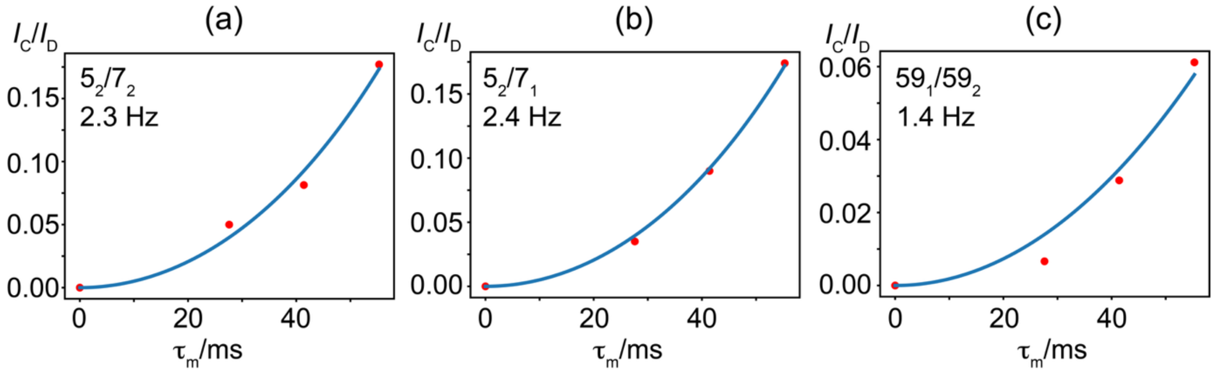

Figure S5. Determination of ${}^{TS}J_{FF}$ couplings by [^{19}F,^{19}F]-TOCSY spectra recorded with different mixing times. The plots show the function $I_C/I_D = \tan^2(\pi J_{FF}\tau_m)$ versus the TOCSY mixing time τ_m for three cross-peaks between CD_2F groups in GB1-d made with the diFLeu version, where all five protons of the isopropyl group are replaced by deuterium (Figure S4). I_C and I_D denote the integrals of, respectively, the cross-peaks and diagonal peaks measured in a one-dimensional cross-section. The cross-peaks are between residues 5 and 7 (a) and (b), and within the solvent-exposed residue 59 (c). Subscripts report the stereospecific assignments of the CD_2F groups (δ_1 or δ_2). The J_{FF} couplings determined from best fits are indicated. The fits were based on the simplifying approximation of a 2-spin system, where the cross-peak intensity grows with $\sin^2(\pi J_{FF}\tau_m)$ and the diagonal peak decays with $\cos^2(\pi J_{FF}\tau_m)$ (Braunschweiler and Ernst, 1983) and the decay by relaxation is cancelled in the ratio I_C/I_D.

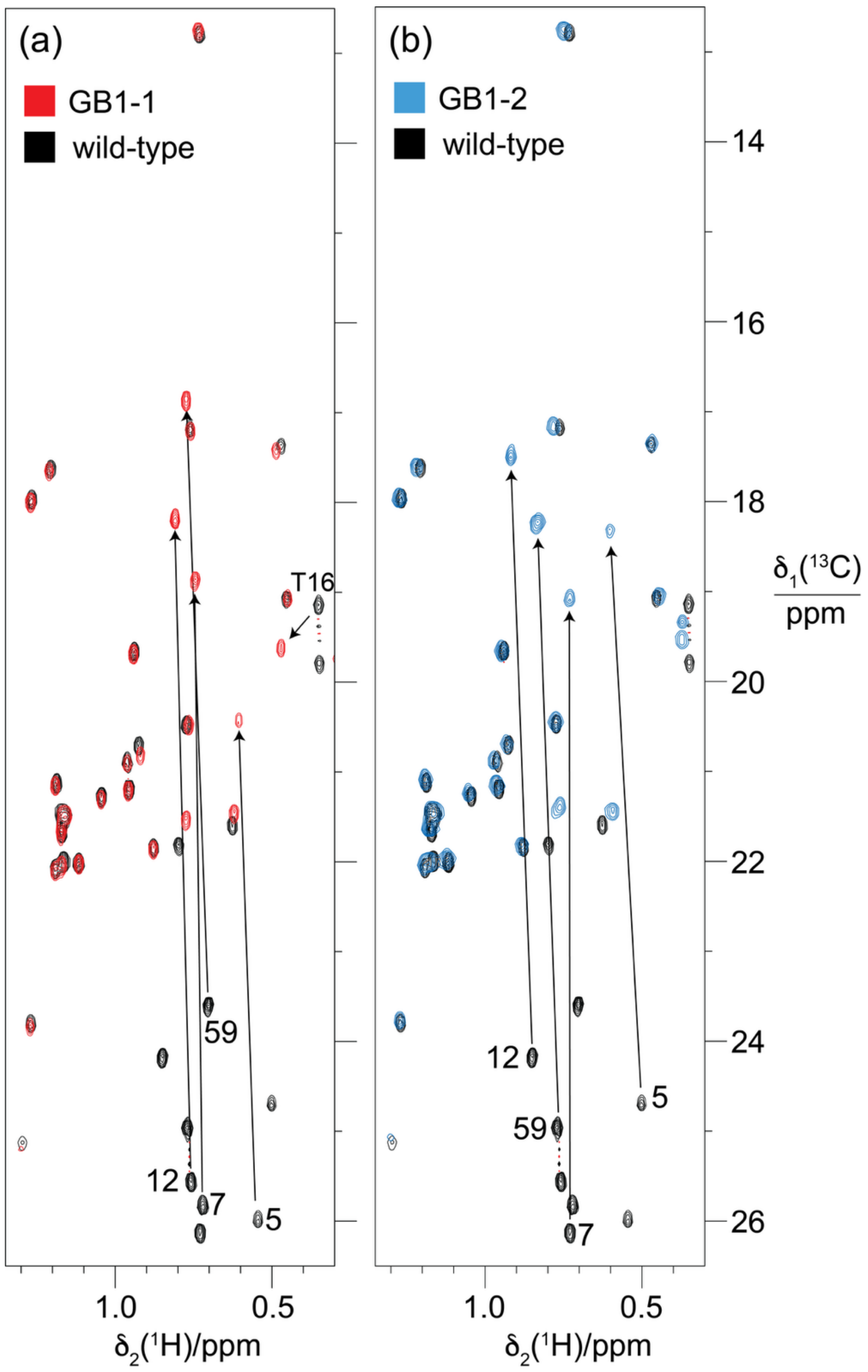

Figure S6. Selected spectral region from the ^{13}C-HSQC spectra of GB1-1 (red) and GB1-2 (blue) superimposed with the spectrum of wild-type GB1 (black). The spectra were recorded at natural isotopic abundance on a Bruker 800 MHz NMR spectrometer equipped with a cryoprobe. The cross-peaks of leucyl residues in wild-type GB1 are labelled with the residue number. Arrows point to the corresponding cross-peaks in GB1-1 (red) and GB1-2 (green). The stereospecific assignments in the wild-type protein were taken from Goehlert et al. (2004). The stereospecific assignment of the CH_3 groups of residue 59 is based solely on the assumption that the change in ^{13}C chemical shift is similar in GB1-1 and GB1-2. (a) ^{13}C-HSQC spectrum of GB1-1, where the change in chemical shifts is caused by FLeu1 residues. The change in

chemical shift for the methyl group of Thr16 can be explained by slightly altered ring currents, as it is near residue 5 and makes NOEs with the aromatic ring protons of Phe30 and Tyr33. Spectrum recorded in MES buffer. (b) ^{13}C-HSQC spectrum of GB1-2, where the change in chemical shifts is due to FLeu2 residues. Spectrum recorded in HEPES buffer.

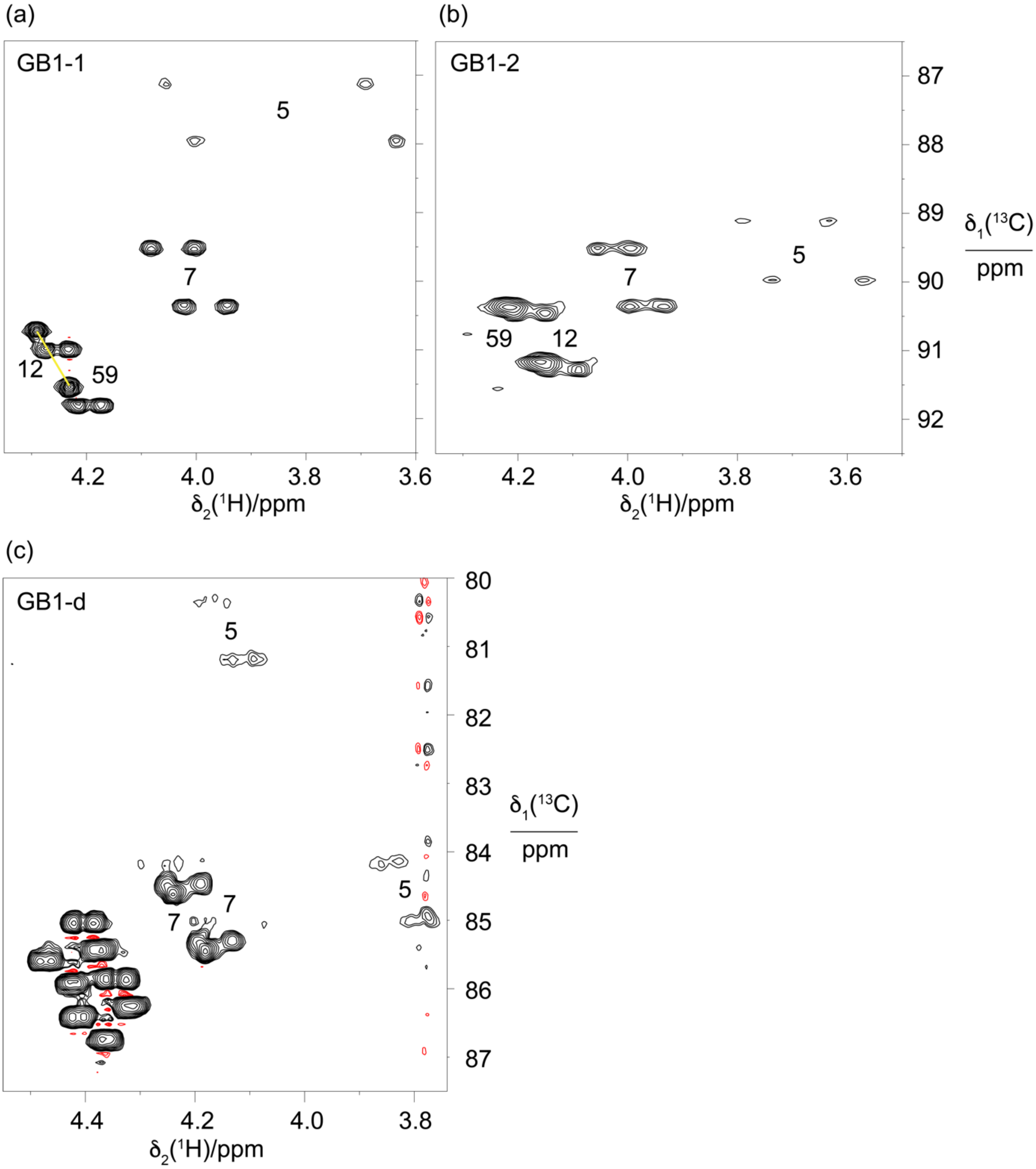

Figure S7. Selected spectral regions from the ^{13}C-HSQC spectra of GB1-1, GB1-2 and GB1-d showing the cross-peaks of the $C^{\delta}H_2F$ groups. The spectra were recorded at natural isotopic abundance on a Bruker 800 MHz NMR spectrometer equipped with a cryoprobe, using t_{1max}

and t_{2max} values of about 24 and 100 ms, respectively, and no ^{19}F decoupling. Cross-peaks are split in the vertical and horizontal dimensions by the $^{1}J_{FC}$ coupling constant (about 163 Hz) and the $^{1}J_{HF}$ coupling constant (about 47 Hz), respectively. The cross-peaks are labelled with the residue number. (a) ^{13}C-^{1}H cross-peaks in GB1-1. A yellow line highlights the multiplet splitting due to the one-bond couplings with ^{19}F in residue 12. The resonances were assigned with the help of cross-peaks with the CH_3 groups in a TOCSY-relayed ^{13}C-HSQC spectrum. Spectrum recorded in MES buffer. (b) ^{13}C-^{1}H cross-peaks in GB1-2. Spectrum recorded in HEPES buffer. (c) ^{13}C-^{1}H cross-peaks in GB1-d. Assignments were obtained by comparison with the short-delay ^{1}H,^{19}F correlation experiment of Figure 9c. Spectrum recorded in MES buffer. Comparison of the chemical shifts of the cross-peaks observed in GB1-d with those in GB1-1 and GB1-2 suggests assignments also for the cross-peaks of residues 12 and 59 not labelled in this figure.

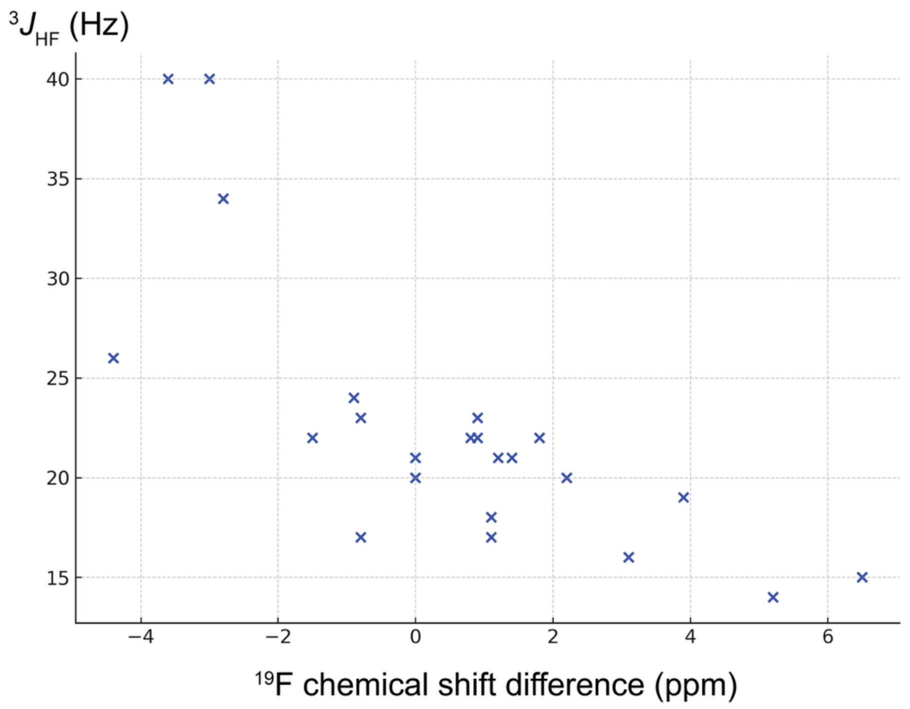

Figure S8. Correlation between $^{3}J_{HF}$ couplings and ^{19}F chemical shifts, illustrating the γ-gauche effect. Chemical shifts are relative to the chemical shifts of the solvent-exposed residue 59. Data taken from Table S4.

Table S1. DNA and corresponding amino acid sequence of the GB1 construct used in the current work.

Protein	DNA sequence	Amino acid sequence[a]
GB1	ATGGCTTCTATGACCGGTATGACCTACAAACTGATC CTGAACGGTAAAACCCTGAAAGGTGAAACCACCACC GAAGCGGTTGACGCGGCGACCGCGGAAAAAGTTTTC AAACAGTACGCGAACGACAACGGTGTTGACGGTGAA TGGACCTACGACGACGCGACCAAAACCTTCACCGTT ACCGAAGAAAACCTGTATTTTCAGGGCCATCATCAT CACCATCAC	MASMTGMTYKLILNGKTLKG ETTTEAVDAATAEKVFKQYA NDNGVDGEWTYDDATKTFTV TEENLYFQGHHHHHH

[a] The present work uses the sequence numbering of wild-type GB1 starting with the second methionine residue. The N-terminal MASMTG tag was numbered -6 to -1.

Table S2. Yields of purified GB1 per mL inner CFPS reaction mixture obtained with fluorinated leucine analogues.

Protein	Yield/mg
GB1 with FLeu1	2.1
GB1 with FLeu2	2.3
GB1 with diFLeu	2.7
GB1 with diFLeu (0.5 mM L-leucine and 4 mM diFLeu)	1.3
GB1 with diFLeu (0.25 mM L-leucine and 4 mM diFLeu)	1.2

Table S3. $R_{1\rho}$(^{19}F) values measured in CPMG experiments with different spacing between 180° pulses and comparison with R_2* values[a]

Sequence position and stereospecific assignment	GB1-d, $R_{1\rho}$ with 1250 Hz CPMG[b]	GB1-d, $R_{1\rho}$ with 31 Hz CPMG[c]	GB1-d-D, $R_{1\rho}$ with 1250 Hz CPMG[d]	GB1-d, R_2* [e]
5, δ_2	31 (1)	37 (3)	15 (1)	53
7, δ_2	15.9 (0.2)	25 (1)	10 (1)	24
12, δ_2	8.3 (0.5)	11.7 (0.2)	3.1 (0.5)	23
12, δ_1	6.8 (0.4)	8.6 (0.1)	3.7 (0.5)	23
59, δ_1	7.3 (0.2)	12.5 (0.7)	4.9 (0.3)	21
59, δ_2	7.8 (0.7)	11.2 (0.4)	3.6 (0.4)	21
7, δ_1	13.6 (0.2)	26 (1)	9.1 (0.4)	29
5, δ_1	37 (1)	33 (4)	20 (1)	56

[a] Measured on a 400 MHz NMR spectrometer. $R_{1\rho}$ and R_2* values given in s^{-1}, showing the standard deviation of fitting in brackets. R_2* values were determined by measuring the full width at half height in 1D ^{19}F-NMR spectra (in Hz) and multiplying by π. GB1-d refers to the samples made with diFLeu residues. GB1-d-D refers to samples made with diFLeu residues, where all five protons of the isopropyl group are replaced by deuterium.

[b] Using 0.4 ms spacing between 180° pulses. Relaxation delays used were 0.8, 16.8, 32.8, 40.8, 56.8, 72.8, 88.8, 104.8 ms.

[c] Using 16 ms spacing between 180° pulses. Relaxation delays used were 32, 48, 64, 80, 96, 112, 128, 144 ms.

[d] Using 0.4 ms spacing between 180° pulses. Relaxation delays used were 0.8, 7.2, 13.6, 20, 26.4, 32.8 39.2, 45.6, 52, 58.4, 64.8, 71.2, 77.6 ms.

[e] 1D ^{19}F-NMR spectrum measured with 2800 Hz GARP decoupling of ^{1}H during 144 ms data acquisition.

Table S4. Measurements of $^{3}J_{HF}$ couplings by short-delay ^{1}H,^{19}F correlation experiments[a]

Residue	^{19}F chemical shift difference (ppm)[b]	Cross-peak intensity ratio[c]	$^{3}J_{HF}$ (Hz)[d]
GB1-1			
12	1.1	11	18
59	0	10	20
7	-0.8	13	17
5	-2.8	4	34
GB1-2			
5	5.2	18	14
12	3.1	15	16
7	1.1	13	17
59	0	9	21
GB1-d			
5	6.5	27	15
7	2.2	12	20
12	1.2	10	21
12	0.9	10	22
59	0.8	9	22
59	-0.8	8	23
7	-1.5	10	22
5	-3.0	3	40
GB1-dd			
5	6.8	> 20	<15
7	3.9	14	19
12	1.8	10	22
12	1.4	11	21
59	0.9	9	23
59	-0.9	8	24
5	-3.6	3	40
7	-4.4	7	26

[a] Based on the spectra shown in Figure 9. A correlation plot is shown in Figure S8.

[b] Difference between the measured chemical shift and the chemical shift of the solvent-exposed residue 59 (average chemical shift of residue 59 in the case of diFLeu samples).

[c] Ratio of the integral measured for the H^{γ}–^{19}F cross-peak versus the combined cross-peak intensities of the $C^{\delta}H_2$–^{19}F cross-peaks.

[d] Calculated as described in the main text.

References

Braunschweiler, L. and Ernst, R. R.: Coherence transfer by isotropic mixing – application to proton correlation spectroscopy, J. Magn. Reson., 53, 512–528, https://doi.org/10.1016/0022-2364(83)90226-3, 1983.

Goehlert, V. A., Krupinska, E., Regan, L., and Stone, M. J.: Analysis of side chain mobility among protein G B1 domain mutants with widely varying stabilities. Prot. Sci., 13, 3322–3330, https://doi.org/10.1110/ps.04926604, 2004.
